# Association of the triglyceride-glucose index/high-density lipoprotein cholesterol ratio, a novel insulin resistance marker, with incident diabetes mellitus: a large-scale retrospective cohort study

**DOI:** 10.3389/fendo.2026.1896239

**Published:** 2026-08-10

**Authors:** Wangsheng Deng, Yonggang Yang, Jie Li, Chuang Gao, Yong Han, Dongna Zhou

**Affiliations:** 1 Department of Emergency Medicine, People’s Hospital of Longhua, Shenzhen, China; 2 Department of Emergency, Shenzhen Dapeng New District Kuichong People’s Hospital, Shenzhen, China; 3 Department of Emergency, Shenzhen Second People’s Hospital, The First Affiliated Hospital of Shenzhen University, Shenzhen, China; 4 Department of Emergency Medicine, The Second Affiliated Hospital of Guangxi Medical University, Nanning, China

**Keywords:** diabetes mellitus, insulin resistance, nonlinear relationship, predictive performance, TyGHR

## Abstract

**Objective:**

Data on the relationship between the triglyceride-glucose index/high-density lipoprotein cholesterol ratio (TyGHR) and diabetes mellitus (DM) onset remain limited. This study investigated the association between TyGHR and incident DM and assessed its predictive utility.

**Methods:**

A total of 8,789 adults who received routine health examinations at Kuichong People’s Hospital, Shenzhen, between 2018 and 2023 were included in this retrospective cohort analysis. The association between TyGHR and incident DM was assessed using Cox proportional hazards models. Potential dose-response patterns were explored using restricted cubic splines. The robustness of the findings was examined through stratified and sensitivity analyses. Conventional receiver operating characteristic (ROC) curves and time-dependent ROC analyses were used to evaluate the predictive performance of TyGHR for incident DM over a follow-up period of up to 5 years.

**Results:**

After adjustment for potential confounders, TyGHR was positively associated with incident DM (HR = 1.108, 95% CI: 1.046–1.173). A nonlinear association was observed, with an estimated statistical threshold of 7.27. Below this threshold, each one-unit increase in TyGHR was associated with a higher risk of incident DM (HR = 1.296, 95% CI: 1.103–1.524). Time-dependent ROC analysis showed relatively consistent predictive performance from 2.0 to 5.0 years, with AUCs ranging from 0.7351 to 0.7402. Sensitivity analyses yielded similar results.

**Conclusion:**

TyGHR was independently associated with incident DM and showed a nonlinear pattern with an estimated threshold of approximately 7.27. It also demonstrated relatively stable predictive capacity over 2 to 5 years of follow-up, suggesting its potential value as a simple indicator for early DM risk stratification and prevention.

## Introduction

Characterized by chronically elevated blood glucose concentrations, diabetes mellitus (DM) represents a heterogeneous group of metabolic disturbances rooted in defective pancreatic insulin output, peripheral insulin resistance, or a combination of both pathological mechanisms (1, 2). Data released through the IDF Diabetes Atlas indicate that the global count of individuals living with DM stood at roughly 425 million in 2017, a number forecast to climb toward 629 million over the subsequent three decades by 2045 (3). Beyond glycemic dysregulation, the disease exerts far-reaching systemic damage—spanning renal, neurological, ophthalmological, and cardiovascular domains—thereby establishing itself among the most consequential drivers of premature disability and death on a global scale (4–10). With both prevalence rates and DM-related mortality continuing their upward trajectory, healthcare infrastructures worldwide face mounting and unprecedented pressure (6, 11). Consequently, identifying risk factors and implementing targeted preventive interventions are of substantial clinical importance for reducing both the incidence of DM and its associated complications.

Insulin resistance (IR) is closely associated with several metabolic disorders, including metabolic syndrome, nonalcoholic fatty liver disease (NAFLD), DM, and obesity (12–15). Since IR is closely related to DM development, its early identification may be important for prevention (12). Although the hyperinsulinemic-euglycemic clamp is considered the reference standard for evaluating IR, its complicated procedure, high cost, and ethical concerns limit its routine clinical use. Thus, a simple, reliable, and reproducible approach is needed for IR assessment (16). The triglyceride-glucose (TyG) index, calculated from fasting plasma glucose (FPG) and triglycerides (TG), has been reported to be a sensitive and specific surrogate marker of IR compared with the clamp method (17). Many studies have shown a positive association between the TyG index and DM incidence (18–20). However, TyG alone has inherent limitations, as it mainly reflects TG-related very-low-density lipoprotein (VLDL) metabolism and does not account for high-density lipoprotein cholesterol (HDL-c) dysfunction (21, 22).Lower HDL-c may reduce apolipoprotein A-I (ApoA-I) availability and impair ATP-binding cassette transporter A1 (ABCA1)-mediated reverse cholesterol transport, thereby aggravating intracellular cholesterol accumulation and promoting IR (23). Consistently, reduced HDL-c has been linked to IR and a higher risk of DM. Therefore, we hypothesize that combining TyG with HDL-c into a composite index may better characterize the underlying pathophysiological process and offer useful information for DM risk assessment and early prediction.

HDL-c has commonly been incorporated as an adjustment component in research on lipid metabolism and metabolic disorders, such as the TG/HDL-c, TC/HDL-c, and non-HDL-c/HDL-c ratios, particularly in studies related to DM and fatty liver disease (24–26). However, limited evidence is available regarding the use of HDL-c to modify the TyG index. Gao et al. recently introduced the HDL-c-adjusted TyG index, also known as the triglyceride-glucose index-to-high-density lipoprotein cholesterol ratio (TyGHR), which showed 92.9% diagnostic accuracy for metabolic dysfunction-associated fatty liver disease (MAFLD) (27). Tong et al. later confirmed the clinical relevance of this ratio for evaluating coronary artery calcification risk, extending its potential application to cardiovascular conditions (28). Nevertheless, the association between TyGHR and DM remains insufficiently investigated. Accordingly, the present retrospective cohort study was designed to assess whether TyGHR is independently associated with incident DM and to evaluate its predictive performance.

## Methods

### Study design and data collection

This retrospective cohort study included adults who underwent routine health examinations at Kuichong People’s Hospital, Dapeng New District, Shenzhen, between January 2018 and December 2023. TyGHR was defined as the exposure variable, and incident DM was defined as the outcome.

Anthropometric assessments were performed by qualified clinical personnel. Baseline data were collected via structured questionnaires, covering lifestyle factors (smoking and alcohol consumption) and sociodemographic and clinical variables including sex, age, and hypertension (HTN). At every health examination, blood pressure was assessed with a standard mercury sphygmomanometer. Venous blood samples were collected after participants had fasted for no less than 10 hours. TG, FPG, LDL-c, and HDL-c were measured using a Beckman 5800 automated analyzer. According to questionnaire responses, alcohol consumption was classified into three categories: never drinkers, defined as participants who did not drink alcohol or consumed fewer than 12 drinks annually; current drinkers, defined as those reporting ongoing alcohol use at any frequency; and ever drinkers, defined as individuals with previous alcohol exposure, including former drinkers and those with intermittent drinking behavior.

### Study population

**Figure 1:**
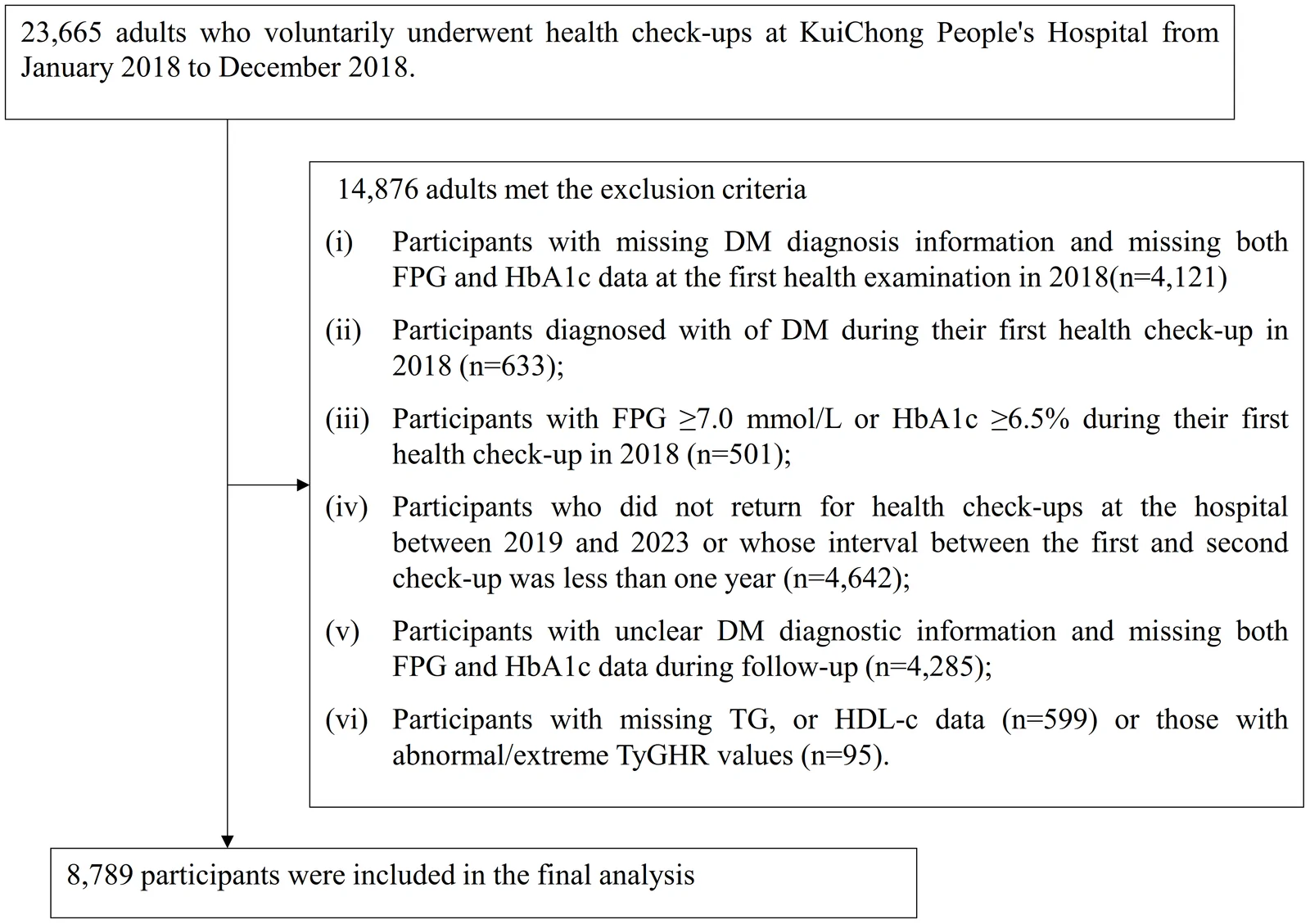
Flowchart illustrates the study participants.

The initial population comprised 23,665 participants aged 18 years or older who underwent routine health check-ups from January to December 2018. Participants were excluded if they met any of the following conditions: (i) absence of DM diagnostic records together with missing fasting plasma glucose (FPG) and glycated hemoglobin (HbA1c) measurements at the baseline visit (n = 4,121); (ii) a diagnosis of DM during the initial examination in 2018 (n = 633); (iii) baseline FPG of 7.0 mmol/L or higher, or HbA1c of 6.5% or higher, in 2018 (n = 501); (iv) no subsequent health examination between 2019 and 2023, or an interval of less than one year between the first and second examinations (n = 4,642); (v) uncertain DM status during follow-up accompanied by unavailable FPG and HbA1c data (n = 4,285); and (vi) missing TG or HDL-c values (n = 599), or abnormal/extreme TyGHR value (n = 95). After these exclusions, 8,789 individuals remained for the final analysis. The selection procedure is presented in **Figure 1**.

### Ethical approval and consent

The study protocol was reviewed and approved by the Ethics Committee of Kuichong People’s Hospital, Dapeng New District, Shenzhen, China (Approval No. 2024005). Because this investigation was retrospective and all data were fully anonymized, the need for written informed consent was waived by the ethics committee. The study was performed in accordance with the Declaration of Helsinki and relevant ethical standards and regulatory requirements.

### The TyG index to HDL-c ratio

TyGHR is calculated as TyGHR = TyG/HDL-c. The TyG value is computed as Ln[FPG (mg/dL) × TG (mg/dL)/2], and HDL-c is expressed in mmol/L (28, 29). When biochemical measurements were recorded in mmol/L, TG and FPG were converted to mg/dL using the following formulas: TG (mg/dL)=TG(mmol/L)×88.57(mg/dL)=TG(mmol/L)×88.57 and FPG (mg/dL)=FPG(mmol/L)×18.0(mg/dL)=FPG(mmol/L)×18.0. Because HDL-c was used in mmol/L for the TyGHR denominator, HDL-c values recorded in mg/dL were converted to mmol/L using the formula: HDL-c (mmol/L) = HDL-c (mg/dL) × 0.02586, equivalent to HDL-c (mg/dL)/38.67.

### Diagnosis of diabetes mellitus and follow-up

Incident DM was identified when participants who were free of DM at baseline received a DM diagnosis during follow-up. DM was determined according to at least one of the following conditions: (1) a self-reported diagnosis made by a physician; (2) FPG level of 7.0 mmol/L or above; or (3) HbA1c level of 6.5% or above (30). At each follow-up assessment, standardized questionnaires were used to obtain information regarding DM diagnosis, diagnostic date, and use of glucose-lowering therapy. For participants in whom DM was identified by laboratory criteria during periodic health examinations, the event date was assigned as follows: if a self-reported physician-diagnosed date was available and preceded the laboratory confirmation date, the self-reported date was used as the event time; if no prior self-reported diagnosis was available and DM was confirmed solely by laboratory findings, the date of the health examination at which the diagnostic criteria were first met was designated as the event date. For those who did not develop DM, follow-up time was measured from baseline until the last available follow-up visit.

### Covariates

Covariates were selected according to clinical relevance and evidence reported in prior research (31–33). Continuous variables comprised body mass index (BMI), C-reactive protein (CRP), age, alanine aminotransferase (ALT), waist circumference (WC), systolic blood pressure (SBP), serum creatinine (Scr), hemoglobin A1c (HbA1c), total cholesterol (TC), gamma-glutamyl transferase (GGT), diastolic blood pressure (DBP), low-density lipoprotein cholesterol (LDL-C), and aspartate aminotransferase (AST). Categorical variables included smoking status, sex, antihyperlipidemic medication use (DLP-MED), hypertension (HTN), drinking status, physical activity, dyslipidemia (DLP), and antihypertensive medication use (HTN-MED).

Two composite insulin resistance indices were defined as follows: TyG-BMI was calculated by multiplying the TyG index by BMI (TyG-BMI = TyG × BMI), where BMI was expressed in kg/m²; and TyG-WC was derived as the product of the TyG index and WC (TyG-WC = TyG × WC), whereWC was measured in centimeters (cm).

### Handling missing data

Observational studies frequently contend with incomplete data. In this analysis, several variables exhibited missing observations. The missing counts and estimated rates are as follows: drinking status (n = 543, 6.18%), smoking status (n = 303, 3.45%), BMI (n = 14, 0.16%), DBP (n = 19, 0.22%), SBP (n = 19, 0.22%), DLP (n = 152, 1.73%), Scr (n = 89, 1.01%), AST (n = 147, 1.67%), ALT (n = 147, 1.67%), WC (n = 93, 1.06%), GGT (n = 89, 1.01%), physical activity (n = 234, 2.66%), and DLP-MED (n = 147, 1.67%). Missing data were addressed using multiple imputation by chained equations (MICE), with 10 iterative cycles performed during the imputation process. The imputation method was chosen according to the nature of each variable. For continuous variables, predictive mean matching (PMM) was applied, which helped retain the original distributional pattern of the data and reduced the likelihood of generating implausible imputed values. For categorical variables, logistic regression models were used for imputation (34, 35). A total of five complete datasets were created, and each dataset was analyzed separately to obtain estimates, including means and regression coefficients (34). The final pooled results were then derived by combining these estimates according to Rubin’s rules. The imputation model incorporated the following predictors: CRP, HTN-MED, BMI, sex, AST, physical activity, Scr, DLP, WC, HbA1c, SBP, smoking status, LDL-c, ALT, age, GGT, DLP-MED, DBP, and HTN. The missingness mechanism was assumed to be Missing at Random (MAR) (34).

### Statistical analysis

Study participants were stratified into four groups based on the quartile distribution of TyGHR (Q1 through Q4), and their clinical and demographic profiles at enrollment were systematically evaluated across strata. Continuous variables with a normal distribution are presented as mean ± standard deviation (SD), whereas skewed variables are presented as median and interquartile range (IQR). Categorical variables are expressed as frequencies and percentages. Differences in continuous variables across TyGHR quartiles were assessed using one-way analysis of variance or the Kruskal–Wallis test, as appropriate. Categorical variables were compared using Pearson’s chi-square test.

To explore the relationship between TyGHR and newly diagnosed DM, Cox proportional hazards regression was applied across three successive model configurations: an unadjusted model, a partially adjusted model, and a fully adjusted model. Prior to model construction, conformity with the proportional hazards assumption was systematically verified through Schoenfeld residual diagnostics, encompassing assessments at both the individual covariate and global model levels. No meaningful departure from this assumption was identified — neither for the primary exposure variable, TyGHR, nor at the aggregate model level — as all P values exceeded 0.05, thereby corroborating the methodological appropriateness of Cox regression within the present analytical framework. Before covariate adjustment, variance inflation factors (VIFs) were also examined for all potential confounders to identify multicollinearity; a VIF value above 5 was regarded as suggestive of meaningful collinearity. Three regression models were developed: Model I was an unadjusted model. Model II included adjustment for age, sex, smoking status, and drinking. Model III was further adjusted for DBP, HTN, LDL-c, Scr, GGT, SBP, CRP, physical activity, HbA1c, DLP-MED, BMI, and ALT based on the adjustments made in Model II. The selection of covariates was based on clinical plausibility and evidence from previous studies. WC and TC were not entered into the final multivariable models because of multicollinearity, as detailed in **Supplementary Table 1**.

Earlier evidence has indicated that HTN, obesity, alcohol intake, and glucose metabolism are closely related (36–38). To examine whether the main results were stable, we performed sensitivity analyses by removing, in sequence, participants with BMI ≥ 28 kg/m², drinkers, and those with HTN (39). In the multivariable Cox proportional hazards models, a generalized additive model (GAM) was further used, in which continuous covariates were fitted as smooth terms. In addition, to assess the potential selection bias introduced by the exclusion criteria, the baseline characteristics of the 8,789 included participants were compared with those of the 14,876 excluded individuals. Furthermore, in this retrospective cohort study, incident DM was ascertained through scheduled health screenings rather than continuous clinical monitoring, precluding precise determination of onset timing and inherently introducing interval censoring. Therefore, a sensitivity analysis was performed by reassigning each event time to the midpoint between the last negative and first positive screening visit, with multivariable Cox models re-estimated using identical covariates. This approach provided an alternative estimate of the event date within the censoring interval. Finally, the E-value was calculated to evaluate the possible effect of unmeasured or unknown confounders on the association between TyGHR and DM risk. The E-value quantifies the strength of association that an unmeasured confounding factor would need to have with both the exposure and the outcome, on the risk-ratio scale, to fully account for the observed exposure–outcome relationship. In the present context, complete attenuation of the observed association would require an unmeasured factor to meet two conditions simultaneously: first, its association with the exposure should be stronger than the calculated E-value, expressed as RR_EU. Second, its association with the outcome(DM) should also exceed the E-value, expressed as RR_UD (40, 41).

Stratified Cox proportional hazards regression was applied for subgroup analyses. The stratification variables included age, SBP, sex, smoking status, DBP, physical activity, and BMI, HTN-MED, DLP. Relevant variables were grouped according to clinically meaningful thresholds. Specifically, age was classified into five categories: <30, 30–40, 40–50, 50–60, and ≥60 years. SBP was dichotomized at 140 mmHg, and DBP was dichotomized at 90 mmHg. In the adjusted models, the covariates included age, BMI, drinking, ALT, physical activity, HbA1c, DBP, smoking status, GGT, HTN, Scr, CRP, DLP-MED, and SBP; however, when a variable served as the stratification factor, it was not entered simultaneously as an adjustment variable. Possible effect modification was examined by comparing models with and without interaction terms using the log-likelihood ratio test.

To evaluate the potential nonlinear relationship between TyGHR and incident DM, a Cox proportional hazards model incorporating restricted cubic splines (RCS) was constructed. Knot locations were specified according to the standard recommendations proposed by Harrell, placed at the 5th, 35th, 65th, and 95th percentiles of the TyGHR distribution. Model fit was compared across specifications of 3, 4, and 5 knots using the Akaike Information Criterion (AIC) and Bayesian Information Criterion (BIC) as model selection criteria. The 4-knot configuration achieved the optimal balance between goodness-of-fit and model parsimony, yielding the lowest values for both AIC and BIC (**Supplementary Table 2**), and was therefore selected for subsequent analyses. The nonlinearity assumption was formally tested using a likelihood ratio test, comparing the full model containing RCS terms against a restricted model including only the linear term of TyGHR, thereby quantifying the incremental contribution of nonlinear components to model fit. If a statistically significant nonlinear association was confirmed, threshold effect analysis was further conducted to quantify the difference in effect estimates on either side of the inflection point. The inflection point was identified using a recursive grid search algorithm: candidate thresholds were evaluated sequentially across the entire observed range of TyGHR values at increments of 0.01 units; at each candidate threshold, a two-piecewise Cox regression model was fitted and the log-likelihood was computed, with the candidate value that maximized the likelihood ratio test statistic defined as the optimal inflection point. To characterize the estimation uncertainty of the identified threshold, bootstrap resampling with 500 iterations was applied to derive the 95% confidence interval (CI). Finally, two independent Cox regression models were fitted separately in subgroups below and above the identified threshold, and a likelihood ratio test was used to confirm the superiority of the piecewise model over the linear model in terms of overall fit.

Receiver operating characteristic (ROC) curve analysis was then performed to compare the ability of TyG-BMI, TyG-WC, TyG, and TyGHR to predict the risk of DM. For each marker, the area under the curve (AUC), best cut-off point, sensitivity, and specificity were estimated. In addition, apparent predictive performance estimated from the training dataset is often subject to optimism bias and may overestimate the true discriminative ability of TyGHR. Therefore, we performed internal validation of the predictive performance of TyGHR using bootstrap resampling with 500 iterations. Furthermore, we used time-dependent ROC curves to evaluate the ability of TyGHR to predict incident DM at prespecified follow-up intervals, including 2, 3, 4, and 5 years. For each time point, the corresponding AUC value, optimal cut-off point, sensitivity, and specificity were estimated. All findings were presented following the STROBE reporting recommendations (42). Statistical processing was completed with R software, version 4.5.3, and Empower, version 5.2. A two-tailed P value below 0.05 was regarded as statistically significant.

## Results

### Characteristics of the participants

**Figure 2:**
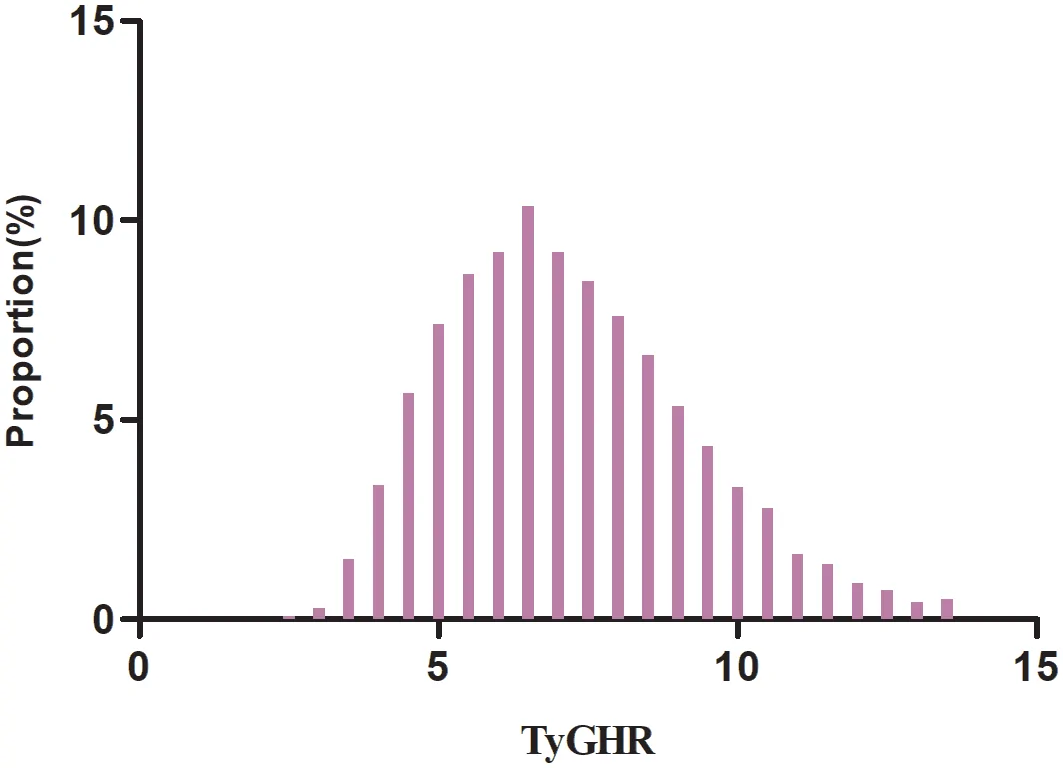
Distribution of TyGHR. The distribution of TyGHR followed a normal pattern, ranging from 2.44 to 13.77, with a mean (SD) of 7.17 (2.16).

The demographic and clinical characteristics of the 8,789 enrolled participants are presented in **Table 1**; the mean age (± standard deviation) was 41.82 ± 8.60, and 63.39% were male. As shown in **Figure 2**, TyGHR was approximately normally distributed, ranging from 2.44 to 13.77, with a mean ± SD of 7.17 ± 2.06. The results of normality and homogeneity-of-variance tests for TyGHR were not statistically significant, further supporting its normal distribution and variance homogeneity (all P > 0.05). Participants were categorized into four groups according to TyGHR quartiles: Q1 (<5.65), Q2 (5.65–6.93), Q3 (6.93–8.47), and Q4 (≥8.47). Compared with those in Q1, participants in the higher TyGHR quartiles were older and had higher levels of GGT, TyG, LDL-c, TyG-BMI, AST, DBP, HbA1c, CRP, WC, ALT, TyG-WC, SBP, BMI, and TC. Moreover, the upper quartile groups had a higher proportion of men and greater prevalences of HTN-MED, DLP, HTN, DLP-MED, and light physical activity.

**Table 1:** Baseline characteristics of participants.

TyGHR quartiles	Q1 (<5.65)	Q2 (5.65-6.93)	Q3 (6.93-8.47)	Q4 (≥8.47)	P-value
Participants(n)	2197	2197	2197	2198	
Age (years)	40.73 ± 8.66	41.84 ± 8.72	42.43 ± 8.62	42.28 ± 8.28	<0.001
DBP (mmHg)	72.78 ± 7.79	75.39 ± 7.64	77.43 ± 7.84	78.80 ± 7.87	<0.001
BMI (kg/m²)	23.96 ± 3.16	25.65 ± 3.50	26.79 ± 3.69	27.89 ± 3.94	<0.001
SBP (mmHg)	111.75 ± 11.75	115.95 ± 11.63	119.21 ± 12.43	121.04 ± 12.45	<0.001
WC (cm)	82.86 ± 10.71	89.86 ± 11.07	94.26 ± 10.83	97.77 ± 10.67	<0.001
TC (mg/dL)	194.32 ± 32.62	195.27 ± 37.18	199.59 ± 38.25	200.57 ± 38.31	<0.001
LDL-c(mg/dL)	110.68 ± 30.67	123.91 ± 33.68	130.23 ± 33.57	128.88 ± 33.35	<0.001
HDL-c (mg/dL)	66.89 ± 10.10	51.17 ± 3.48	43.40 ± 2.88	35.06 ± 3.73	<0.001
TG (mg/dL)	83.78 ± 33.50	101.32 ± 38.82	129.94 ± 52.21	186.71 ± 89.94	<0.001
FPG (mg/dL)	83.33 ± 7.88	86.34 ± 8.11	88.00 ± 8.37	89.80 ± 8.88	<0.001
Scr (umol/L)	69.00 ± 15.21	70.12 ± 15.33	69.88 ± 15.17	70.88 ± 15.73	0.131
TyG index	8.08 ± 0.39	8.31 ± 0.39	8.57 ± 0.41	8.94 ± 0.44	<0.001
TyG-WC	670.56 ± 98.51	748.12 ± 107.53	809.24 ± 109.34	874.79 ± 112.52	<0.001
TyG-BMI	193.90 ± 29.15	213.52 ± 33.26	230.03 ± 35.93	249.59 ± 39.56	<0.001
AST (U/L)	26.83 ± 11.76	28.46 ± 9.07	30.14 ± 12.71	31.66 ± 11.95	<0.001
CRP (mg/L)	1.00 (0.40-2.20)	1.03 (0.50-2.10)	1.20 (0.60-2.40)	1.50 (0.80-2.80)	<0.001
ALT (U/L)	28.00 (22.00-36.00)	30.00 (25.00-35.00)	34.00 (27.00-43.00)	38.00 (30.00-50.00)	<0.001
HbA1c(%)	4.89 ± 0.23	4.98 ± 0.24	5.03 ± 0.25	5.08 ± 0.26	<0.001
GGT (U/L)	20.00 (16.00-28.00)	25.00 (18.00-36.00)	29.00 (22.00-41.00)	33.00 (25.00-47.00)	<0.001
HTN, n (%)	128 (5.83%)	184 (8.38%)	254 (11.56%)	320 (14.56%)	<0.001
DLP, n (%)	385 (17.52%)	535 (24.35%)	670 (30.50%)	812 (36.94%)	<0.001
Sex n (%)					<0.001
Female	1281 (58.31%)	566 (25.76%)	758 (34.50%)	613 (27.89%)	
Male	916 (41.69%)	1631 (74.24%)	1439 (65.50%)	1585 (72.11%)	
DLP-MED, n (%)	124 (5.64%)	204 (9.29%)	219 (9.97%)	220 (10.01%)	<0.001
HTN-MED, n (%)	126 (5.74%)	184 (8.38%)	256 (11.65%)	314 (14.29%)	<0.001
Smoking, n (%)	128 (5.83%)	156 (7.10%)	174 (7.92%)	222 (10.10%)	<0.001
Physical activity, n (%)					<0.001
Sedentary	412 (18.75%)	418 (19.03%)	468 (21.30%)	601 (27.34%)	
Light activity	788 (35.87%)	803 (36.55%)	897 (40.83%)	846 (38.49%)	
Moderate activity	733 (33.36%)	758 (34.50%)	682 (31.04%)	617 (28.07%)	
Vigorous activity	264 (12.02%)	218 (9.92%)	150 (6.83%)	134 (6.10%)	
Drinking n (%)	191 (8.69%)	501 (22.80%)	807 (36.73%)	1139 (51.82%)	<0.001

Normally distributed continuous variables are expressed as mean ± SD, whereas those exhibiting skewed distributions are characterized by the median and IQR. Categorical variables are described using absolute frequencies and their corresponding proportions. DLP-MED, Dyslipidemia Medication; TyG, Triglyceride-glucose index; HbA1c, Hemoglobin A1c; TyG-BMI, Triglyceride-glucose body mass index; TyG-WC, Triglyceride-glucose waist circumference; WC, Waist Circumference; AST, Aspartate Aminotransferase; HTN, Hypertension; TyGHR, Triglyceride-glucose index-to-HDL-c ratio; Scr, Serum Creatinine; TC, Total Cholesterol; ALT, Alanine Aminotransferase; N, Number; FPG, Fasting Plasma Glucose; DLP, Dyslipidemia; HDL-c, High-Density Lipoprotein cholesterol; BMI, Body Mass Index; GGT, Gamma-Glutamyl Transferase; HTN-MED, Hypertension Medication; LDL-c, Low-Density Lipoprotein cholesterol; CRP, C-Reactive Protein; TG, Triglycerides.

### Incidence of DM

A total of 8,789 individuals were enrolled in the present analysis, with a median observation period of about 3.05 years. When participants were grouped according to TyGHR quartiles, the incidence rates of DM were 75.25, 112.68, 181.46, and 237.25 per 10,000 person-years from Q1 to Q4, respectively. Over the follow-up period, 317 participants developed incident DM, corresponding to an overall cumulative incidence of 3.61%. The cumulative incidence increased progressively across TyGHR categories, from 1.78% in Q1 to 2.64% in Q2, 4.28% in Q3, and 5.73% in Q4. Compared with individuals in the lowest TyGHR quartile, those in the highest quartile had a substantially greater incidence of DM, with a significant dose–response trend across quartiles (P for trend < 0.001; **Table 2**).

**Table 2:** Incidence rate of DM (% or Per 10000 person-year).

TyGHR quartiles	Participants (n)	DM events (n)	Incidence rate (95% CI) (%)	Per 10,000 person-year
Total	8789	317	3.61 (3.22–4.00)	152.25
Q1	2197	39	1.78 (1.22–2.33)	75.25
Q2	2197	58	2.64 (1.97–3.31)	112.68
Q3	2197	94	4.28 (3.43–5.12)	181.46
Q4	2198	126	5.73 (4.76–6.71)	237.25
P for trend			<0.001	

CI, confidence; n, number.

**Figure 3:**
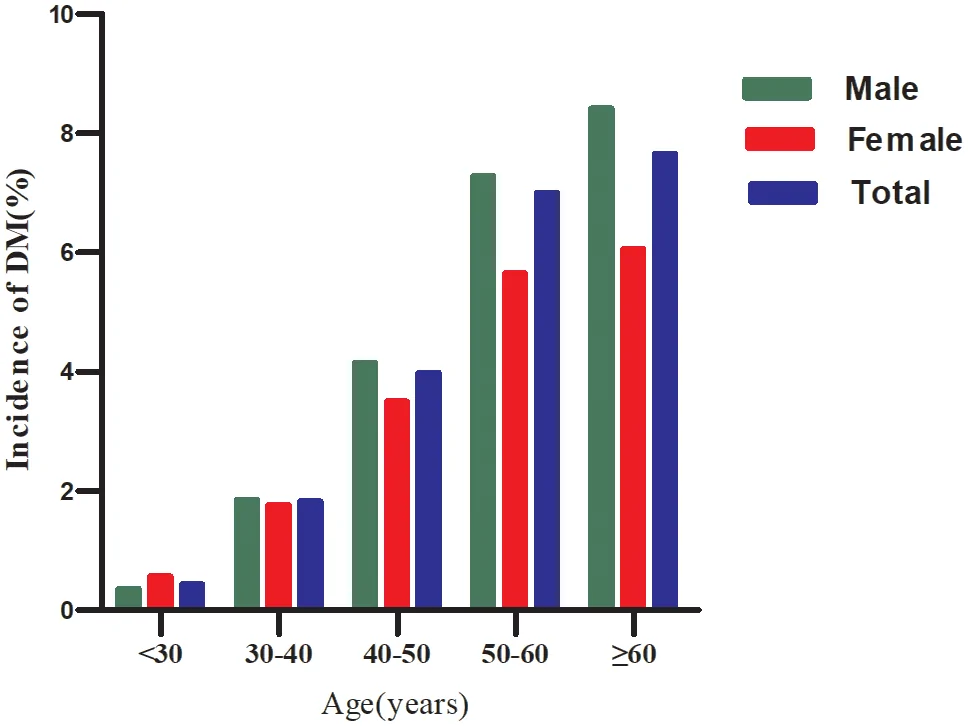
Diabetes mellitus incidence (%) stratified by age and sex.

**Figure 4:**
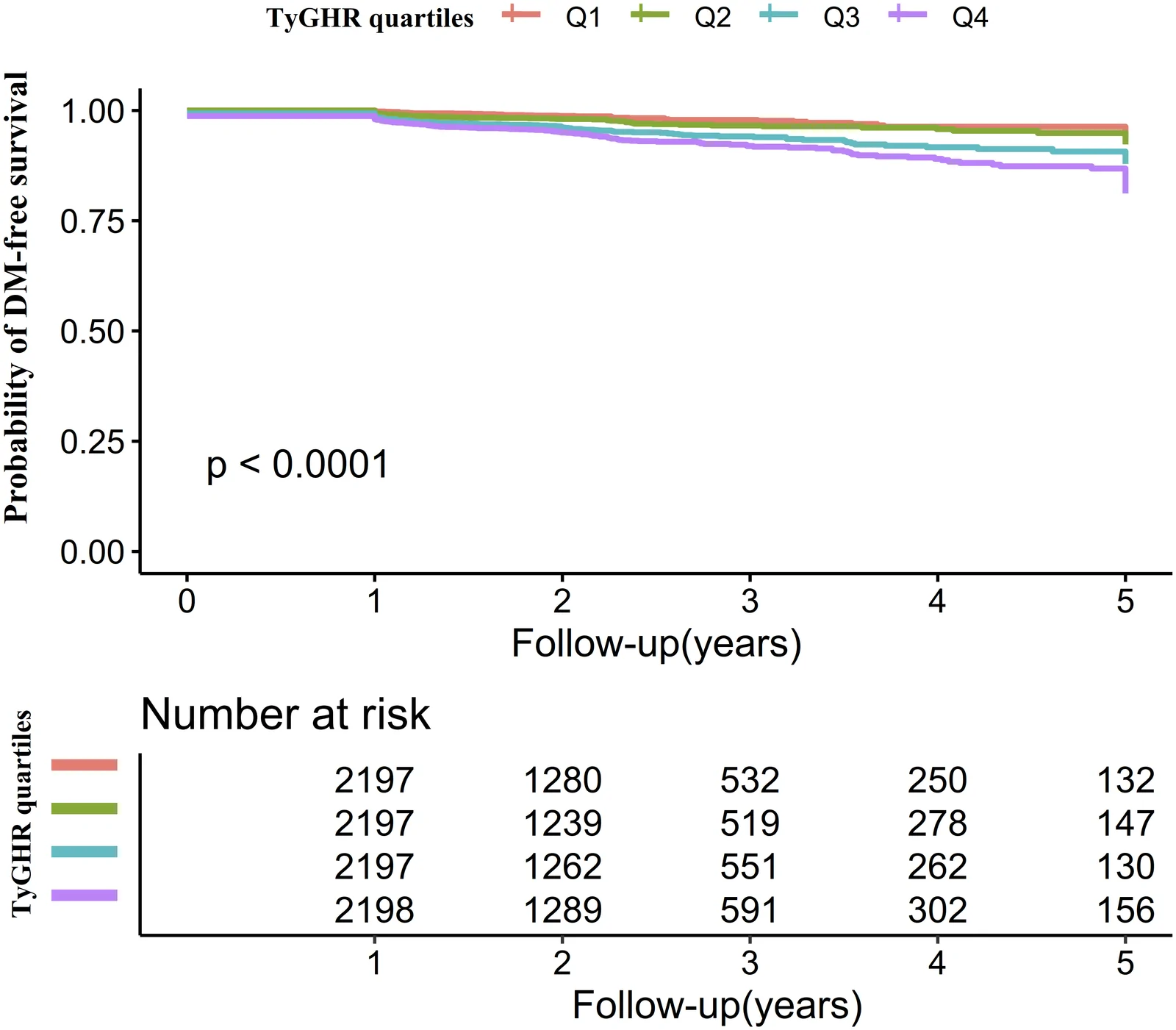
Kaplan-Meier curves illustrating DM–free survival probabilities across TyGHR quartiles.

Participants were further classified into four age categories: <30, 30–40, 40–50, and ≥50 years. As presented in **Figure 3**, men consistently showed a higher probability of developing DM than women within each age stratum. In both sexes, DM incidence rose gradually with advancing age. Kaplan–Meier curves generated according to TyGHR quartiles demonstrated clear separation in DM-free survival among the four groups, and the between-group difference was statistically significant by the log-rank test (P < 0.001; **Figure 4**). The survival analysis further suggested that individuals in the highest TyGHR quartile experienced the greatest risk of incident DM.

### The relationship between TyGHR and the risk of DM

The relationship between TyGHR and DM risk was examined through three hierarchically structured Cox proportional hazards regression models. In the unadjusted framework (Model I), each one-unit rise in TyGHR corresponded to a 19.5% elevation in DM risk (HR = 1.195, 95% CI: 1.138–1.254). Upon controlling for demographic and behavioral characteristics — namely age, sex, smoking status, and alcohol consumption — Model II demonstrated that the association persisted, with every unit increment in TyGHR conferring an 11.8% greater likelihood of DM development (HR = 1.113, 95% CI: 1.055–1.183). Even after further incorporation of an extended panel of clinical and biochemical parameters, TyGHR retained a significant and independent positive relationship with DM incidence in the fully adjusted model (HR = 1.108, 95% CI: 1.046–1.173), as detailed in **Table 3**.

**Table 3:** Relationship between TyGHR and DM risk in different models.

Exposure	Model I (HR,95%CI) P	Model II (HR,95%CI) P	Model III (HR,95%CI) P	Model IV (HR,95%CI) P
TyGHR (Per 1-unit)	1.195 (1.138–1.254) <0.001	1.118 (1.055, 1.183) <0.001	1.108 (1.046–1.173) <0.001	1.082 (1.020–1.147) 0.008
TyGHR (quartiles)				
Q1	Ref	Ref	Ref	Ref
Q2	1.488 (0.992–2.234) 0.055	1.240 (0.817–1.883) 0.312	1.207 (0.796–1.832) 0.375	1.111 (0.729–1.693) 0.623
Q3	2.411 (1.660–3.502) <0.001	1.717 (1.147–2.571) 0.009	1.636 (1.091–2.453) 0.017	1.463 (0.972–2.203) 0.068
Q4	3.099 (2.164–4.439) <0.001	1.983 (1.324–2.969) <0.001	1.927 (1.287–2.886) 0.001	1.632 (1.082–2.461) 0.019
P for trend	<0.001	<0.001	<0.001	0.006

CI: confidence, Ref: reference; HR, Hazard ratios.

Model I: we did not adjust other covariates.

Model II: we adjust sex, age smoking status and drinking status.

Model III: we adjust age, drinking status, sex, BMI, ALT, LDL-c, physical activity, HbA1c, DBP, smoking status, Scr, CRP, GGT, DLP-MED, HTN, and SBP.

Model IV: we adjusted BMI(smooth), age(smooth), sex,ALT(smooth), drinking status, LDL-c(smooth), physical activity, HbA1c(smooth), DBP(smooth), Scr(smooth), smoking status, CRP(smooth), HTN, GGT(smooth), DLP-MED, and SBP(smooth).

CI, confidence interval; HR, hazard ratio; Ref, reference.

Additionally, TyGHR was re-entered into regression analyses after stratifying by quartiles. After taking the first quartile as the reference group in the fully adjusted Cox model, the estimated HRs for DM were 1.207 for Q2 (95% CI: 0.796–1.832), 1.636 for Q3 (95% CI: 1.091–2.453), and 1.927 for Q4 (95% CI: 1.287–2.886). Although the risk estimate for Q2 suggested a modest increase, this association did not reach statistical significance. In contrast, participants in Q3 and Q4 had significantly elevated risks of developing DM, corresponding to approximately 63.6% and 92.7% higher risks, respectively, compared with those in Q1. (see **Table 3**, Model III). Overall, the risk of DM showed a general upward trend from Q1 to Q4(P < 0.001), which is consistent with the results of the analysis of TyGHR as a continuous variable.

### Sensitivity analyses

The robustness of our results was examined through several sensitivity analyses. In the first, smooth spline functions were applied to continuous covariates within the Cox regression framework using a generalized additive model (GAM), allowing us to evaluate whether non-linear adjustments altered the relationship between TyGHR and DM risk. The results indicated that each one-unit rise in TyGHR corresponded to a roughly 8.2% greater risk of DM (HR = 1.082, 95% CI: 1.020–1.147), a result closely mirroring that of the fully adjusted Model III (**Table 3**, Model IV).

The consistency of the observed association was further assessed in several restricted populations. After multivariable adjustment, TyGHR remained positively related to incident DM among participants with a BMI below 28 kg/m²; specifically, a 1-unit increment in TyGHR was linked to a 9.3% higher DM risk (HR = 1.093, 95% CI: 1.021–1.170; **Table 4**). A similar pattern was found after removing individuals with HTN from the analysis, with the corresponding HR estimated at 1.091 for each unit increase in TyGHR (95% CI: 1.020–1.168). In addition, when the cohort was limited to participants who did not consume alcohol, the association persisted and remained statistically significant (HR = 1.132, 95% CI: 1.020–1.256; **Table 4**). These findings support the robustness of the main results across different analytic subsets.

**Table 4:** Association of TyGHR with DM risk in different sensitivity analyses.

Exposure	Model I HR (95% CI) P	Model II HR (95% CI) P	Model III HR (95% CI) P
TyGHR (Per 1-unit)	1.093 (1.021, 1.170) 0.011	1.091 (1.020–1.168) 0.011	1.132 (1.020, 1.256) 0.009
TyGHR (quartiles)			
Q1	Ref	Ref	Ref
Q2	1.179 (0.743, 1.870) 0.485	1.207 (0.758–1.922) 0.428	1.278 (0.966, 1.692) 0.137
Q3	1.591 (1.012, 2.502) 0.044	1.673 (1.063–2.634) 0.026	1.410 (1.035, 1.923) 0.029
Q4	1.719 (1.086, 2.720) 0.021	1.763 (1.111–2.796) 0.016	1.912 (1.056, 3.461) 0.032
P for trend	0.009	0.007	0.019

Model I was a sensitivity analysis among subjects having BMI values under 28 kg/m² (n=7,569). HbA1c, sex, smoking status, DBP, DLP-MED, age, CRP, SBP, physical activity, Scr, BMI, GGT, drinking status, HTN, ALT, and LDL-c adjusted.

Model II was a sensitivity analysis following the exclusion of HTN individuals (N = 7,903). HbA1c, sex, smoking status, DBP, DLP-MED, age, CRP, SBP, physical activity, Scr, BMI, GGT, drinking status, ALT, and LDL-c were adjusted.

Model III was used for sensitivity analysis after excluding drinking participants (N = 6,151) and was adjusted for HbA1c, sex, DBP, DLP-MED, age, CRP, SBP, physical activity, Scr, BMI, GGT, HTN, ALT, and LDL-c.

A sensitivity analysis was additionally performed in which the event time was redefined as the midpoint between the last normal health examination date and the first abnormal health examination date, followed by re-estimation of the multivariable Cox regression model. This approach yielded a more conservative estimate of the event date within the censoring interval. The association between TyGHR and DM remained statistically significant (HR = 1.067, 95% CI: 1.007–1.130), consistent with the primary analysis (**Supplementary Table 3**). In addition, to assess potential selection bias introduced by the exclusion criteria, baseline characteristics were compared between included and excluded participants. Baseline characteristics were generally comparable between included and excluded participants, although minor differences were observed for some variables. These findings suggest that major selection bias due to the exclusion criteria was unlikely, but could not be completely excluded (**Supplementary Table 4**). The potential impact of residual or unmeasured confounding was evaluated using an E-value approach. For the main association observed between TyGHR and incident DM, with a multivariable-adjusted HR of 1.108 and a 95% CI of 1.046–1.173, the corresponding E-value was 1.45. In the present analysis, the estimated association between the unmeasured confounder and DM was stronger than this benchmark value (RR_UD = 1.65 vs. E-value = 1.45). However, the assumed association between the same unmeasured factor and TyGHR exposure was weaker than the E-value threshold (RR_EU = 1.33), suggesting that the observed relationship was unlikely to be fully explained by unmeasured confounding. The aforementioned sensitivity analyses collectively substantiate the stability and reliability of the findings in this study.

### Subgroup analyses

Across both prespecified and hypothesis-generating stratified analyses, TyGHR showed no statistically significant interaction with any of the examined variables, including age, sex, SBP, physical activity, DBP, HTN-MED, DLP, BMI, or smoking status (all P for interaction > 0.05). Collectively, these findings indicate that none of the above factors meaningfully modified or attenuated the relationship between TyGHR and the risk of DM (**Supplementary Table 5**).

### Nonlinear association between TyGHR and incident DM

**Figure 5:**
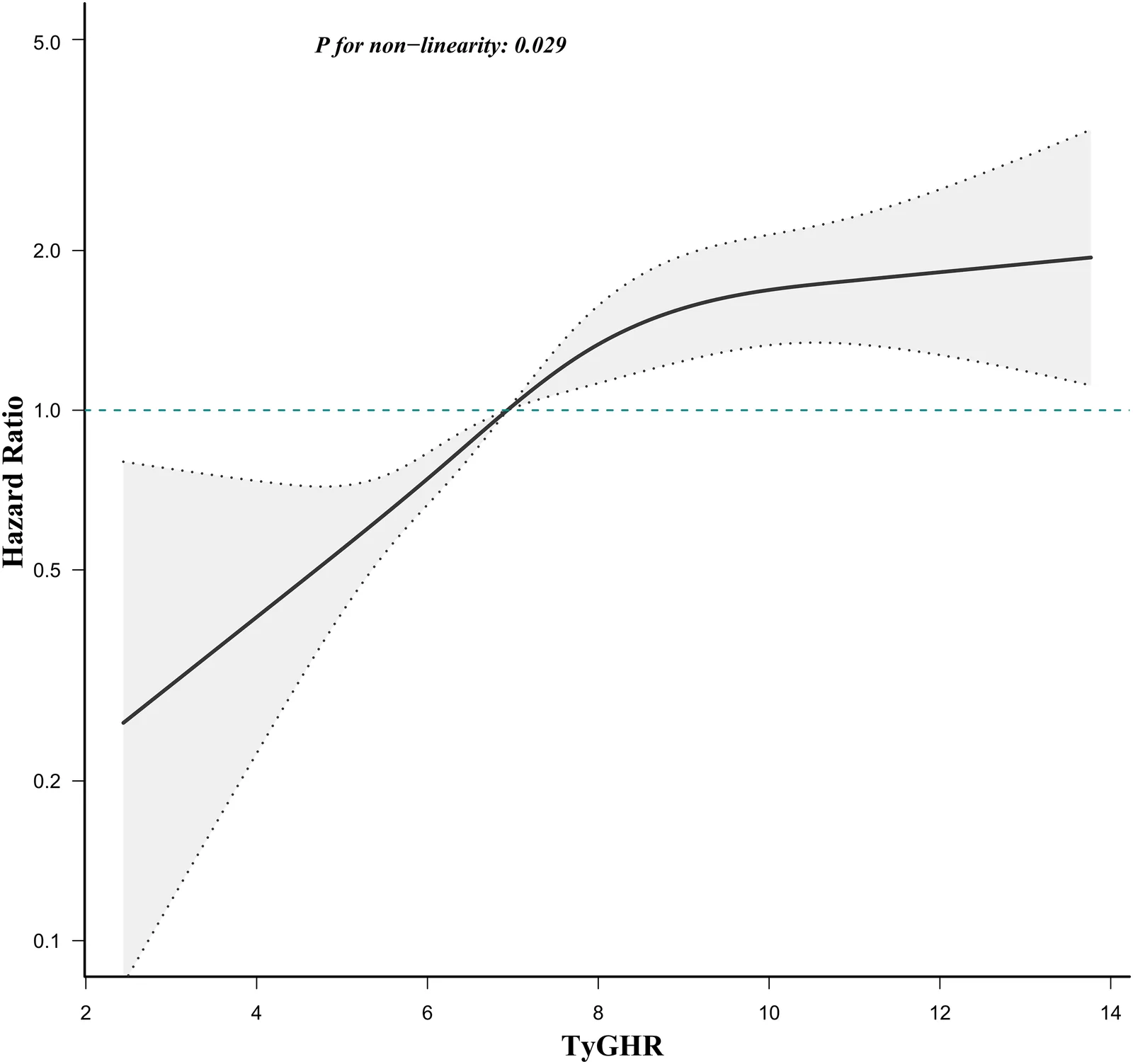
Nonlinear relationship between TyGHR and DM risk.

Restricted cubic spline analysis incorporated into the Cox proportional hazards model indicated a significant nonlinear association between TyGHR and the risk of developing DM, as supported by the test for nonlinearity (P< 0.001; **Figure 5**). By applying a recursive procedure combined with grid-search estimation, the turning point of this relationship was located at a TyGHR level of 7.27. The robustness of this threshold was further supported by bootstrap validation with 500 resampling cycles, yielding a 95% CI of 6.83–7.81. A two-piece Cox regression model was fitted to estimate HRs and corresponding 95% CIs below and above the identified breakpoint. For individuals with TyGHR values lower than 7.27, each 1-unit increase in TyGHR was associated with a significantly higher risk of DM (HR = 1.296, 95% CI: 1.103–1.524). However, beyond this threshold, the association was attenuated and no longer statistically significant (HR = 1.044, 95% CI: 0.962–1.133; **Table 5**).

**Table 5:** Results of the two-piecewise Cox regression model.

Outcome: DM	HR (95% CI) P
Inflection point of TyGHR	7.27 (6.83-7.81)
< 7.27	1.296 (1.103, 1.524) 0.002
≥7.27	1.044 (0.962, 1.133) 0.302
P for log-likelihood ratio test	0.037

Adjusted for age, sex, BMI, smoking status, drinking status, physical activity, ALT, LDL-c, HbA1c, DBP, SBP, Scr, CRP, GGT, DLP-MED, and HTN. CI, confidence interval; HR, hazard ratio.

### ROC analysis of the predictive value of TyGHR, TyG, TyG-BMI, and TyG-WC for DM risk

**Figure 6:**
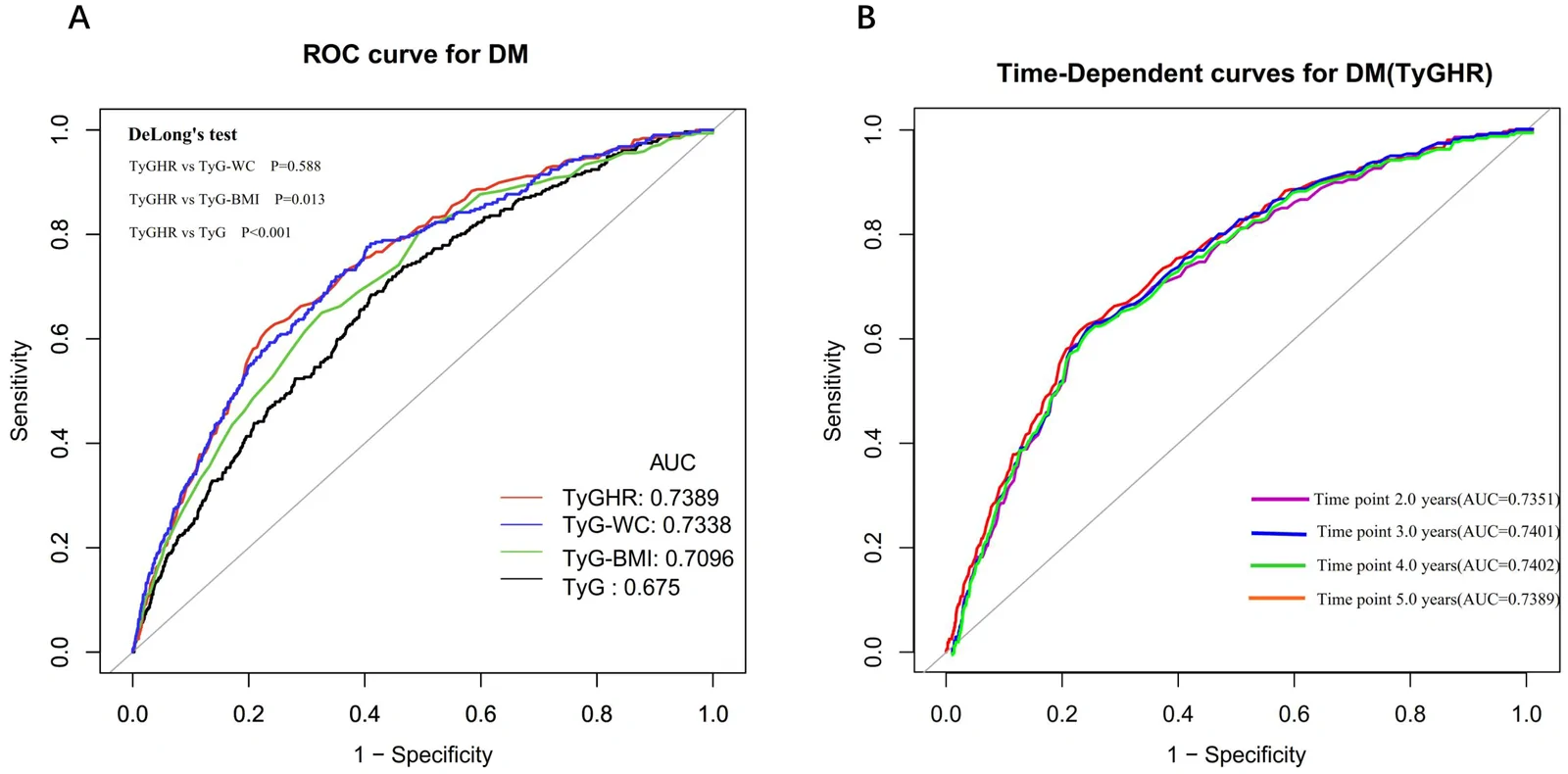
ROC and time-dependent ROC curves for predicting incident diabetes mellitus. **(A)** ROC curves of TyGHR, TyG-WC, TyG, and TyG-BMI for predicting incident diabetes mellitus. **(B)** Time-dependent ROC curves of TyGHR.

To evaluate the discriminative ability of TyGHR alongside TyG, TyG-BMI, and TyG-WC in predicting future DM occurrence, ROC curve analysis was performed (**Figure 6A**). The AUCs ranked from lowest to highest were as follows: TyG (0.6754), TyG-BMI (0.7096), TyG-WC (0.7338) and TyGHR (0.7389). Corresponding optimal cutoff values were 8.571 for TyG, 231.691 for TyG-BMI, 800.107 for TyG-WC, and 6.560 for TyGHR, yielding Youden indices of 0.274, 0.324, 0.373, and 0.385, respectively (**Table 6**). These findings indicated that TyGHR had the highest Youden index and AUC among the compared indices and showed higher discriminative performance than TyG-BMI and TyG for incident DM (DeLong’s test, all P < 0.05). The predictive capacity of TyGHR for incident DM was comparable to that of TyG-WC, with no statistically significant difference observed between the two indices (DeLong’s test, P > 0.05). Additionally, internal validation of the predictive performance of TyGHR was performed using bootstrap resampling with 500 iterations. The bootstrap-validated AUC was 0.7301 (95% CI: 0.7032–0.7566), which was highly consistent with the apparent AUC of 0.7389 (**Supplementary Figure 1**). These findings suggest that TyGHR has good internal validity and that its predictive performance was not substantially overestimated due to overfitting.

**Table 6:** Predictive performance of TyGHR, TyG, TyG-BMI, and TyG-WC for incident DM.

Test	AUC (95% CI)	Best threshold	Sensitivity	Specificity	Youden index
TyGHR	0.7389 (0.7116-0.7661)	6.560	0.770	0.615	0.385
TyG	0.6754 (0.6463-0.7046)	8.571	0.685	0.589	0.274
TyG-WC	0.7338 (0.7057-0.7619)	800.107	0.597	0.776	0.373
TyG-BMI	0.7096 (0.6809-0.7383)	231.691	0.674	0.6498	0.324
TyGHR (Bootstrap 500)	0.7301 (0.7032-0.7566)	6.610	0.776	0.607	0.383

TyGHR, triglyceride-glucose index-to-HDL-c ratio; TyG, triglyceride-glucose index; TyG-BMI, triglyceride-glucose body mass index; TyG-WC, triglyceride-glucose waist circumference; HDL-c, high-density lipoprotein cholesterol. TyGHR (Bootstrap 500) indicates the internally validated predictive performance of TyGHR based on 500 bootstrap resampling iterations.

Decision curve analysis further suggested that TyGHR may have potential clinical utility in DM risk prediction. Across a wide range of threshold probabilities, the TyGHR-based model yielded greater net benefit compared with both the “treat-all” and “treat-none” strategies, suggesting that this index may provide potential clinical net benefit for DM risk stratification across selected threshold probabilities (**Supplementary Figure 2**). Furthermore, the calibration curve showed generally acceptable agreement between predicted and observed risks, suggesting no obvious evidence of poor calibration (**Supplementary Figure 3**).

### Time-dependent ROC analysis of the predictive value of TyGHR for DM risk

The prognostic utility of TyGHR for DM across heterogeneous surveillance windows was further interrogated via time-dependent ROC analysis. At 2, 3, 4, and 5 years of follow-up, the corresponding AUC values for new-onset DM were 0.7351 (95% CI: 0.6994–0.7708), 0.7401 (95% CI: 0.7101–0.7701), 0.7402 (95% CI: 0.7117–0.7687), and 0.7389 (95% CI: 0.7116–0.7661), respectively. These findings suggest that TyGHR maintains a relatively consistent predictive capability for the onset of DM across both shorter and longer follow-up periods (as shown in **Figure 6B** and **Supplementary Table 6**).

## Discussion

This study demonstrates an independent positive association between TyGHR and DM risk. Additionally, a saturable dose–response relationship was observed, with an inflection point at TyGHR of 7.27; the strength of the association differed on either side of this threshold. Moreover, ROC curve analyses showed that TyGHR had a higher AUC than TyG and TyG-BMI and a comparable AUC to TyG-WC, suggesting moderate discriminative ability for incident DM.

Several large-scale cohort studies have validated the utility of the TyG index as a reliable and reproducible composite surrogate for IR (43–45). One large-scale study enrolling over 200,000 subjects demonstrated that, following comprehensive multivariable adjustment, elevated TyG independently predicted DM onset (HR = 3.34, 95% CI: 3.11–3.60) (43). This association has been further corroborated in Chinese populations: among 15,012 adults with prediabetes, those in the upper quartile of TyG exhibited markedly greater DM incidence compared with the lowest quartile (HR = 2.03, 95% CI: 1.71–2.40) (44). Similarly, data derived from the CHARLS cohort, comprising 6,258 individuals aged 45 years or older, revealed a robust and statistically significant dose-dependent relationship between TyG and subsequent DM development (HR = 1.75, 95% CI: 1.56–1.97) (46). In addition, studies confirm that HDL-c is an important regulator of glucose–lipid metabolism. From a lipid metabolic mechanism perspective, there is a significant negative correlation between HDL-c levels and IR (22); decreased HDL-c is accompanied by impaired HDL function, manifested as reduced cholesterol efflux capacity, diminished anti-inflammatory and antioxidant activities, and changes in HDL particle size and protein composition, collectively accelerating the onset and progression of IR (23, 47, 48). Conversely, increasing HDL-c is expected to improve insulin sensitivity (48). A large body of evidence shows a significant negative association between HDL-c and DM risk (49–51). Given the important roles of TyG index and HDL-c in DM pathogenesis, integrating the two into a composite index may more comprehensively capture the pathophysiological features of metabolic dysregulation, thereby providing a more effective biomarker for DM risk assessment and early prediction. In recent years, several modified TyG indices have been reported (e.g., TyG-BMI, TyG-WC); however, studies using HDL-c to adjust TyG remain relatively scarce.

TyGHR, an HDL-c–modified form of TyG recently introduced by Gao et al., integrates HDL-c with the TyG index (27). In their analysis, after controlling for relevant covariates, a 1-unit increment in TyGHR corresponded to markedly higher odds of MAFLD prevalence (OR = 3.162, 95% CI: 1.228–8.141, P = 0.017), and its AUC for identifying MAFLD was 0.929 (27). Evidence from another Chinese cohort of 2,392 individuals with acute decompensated heart failure also suggested prognostic relevance. In multivariable Cox models, each 1-SD rise in TyGHR was linked to a 24% elevation in 30-day all-cause mortality (HR = 1.24, 95% CI: 1.12–1.36) and a 20% increase in cardiovascular death (HR = 1.20, 95% CI: 1.08–1.35) (52). Moreover, elevated TyGHR has been reported to correlate independently with a greater likelihood of coronary calcification (OR = 1.14, 95% CI: 1.05–1.23; OR = 1.15, 95% CI: 1.05–1.21) (28). However, evidence regarding the association between TyGHR and DM remains limited. In the present study, TyGHR was independently associated with incident DM. TyGHR was analyzed both as a continuous variable and by quartiles, allowing assessment of overall and dose-related associations with DM risk. Sensitivity analyses restricted to participants with BMI < 28 kg/m², those without HTN, and non-drinkers yielded consistent results, supporting the robustness of the observed association across different subgroups. To address potential selection bias arising from the relatively large number of excluded participants, the baseline characteristics of the 8,789 included participants were compared with those of the 14,876 excluded individuals, revealing no clinically meaningful differences in demographic or metabolic variables. Notably, most exclusions were attributable to administrative or data quality reasons—specifically, missing laboratory data, insufficient follow-up duration, or unclear DM status during follow-up—rather than selective exclusion based on clinical risk profiles. The missing data mechanism therefore more closely approximates missing at random than informative exclusion, suggesting a limited risk of systematic selection bias. Elucidating the association between TyGHR and DM risk may have clinical implications. In clinical practice, incorporating TyGHR into routine metabolic assessment systems may help clinicians identify individuals at higher risk of developing DM and support earlier risk stratification. However, because the present study was observational and retrospective in nature, these findings should not be interpreted as evidence that lowering TyGHR would necessarily reduce DM incidence. Rather, TyGHR may serve as a readily available supplementary marker for identifying individuals who may benefit from closer metabolic monitoring and lifestyle assessment. At the public health level, individuals with elevated TyGHR may represent a population in whom established preventive strategies, such as increased physical activity and improved dietary patterns, deserve greater attention.

Similarly, the present findings demonstrated that TyGHR showed a higher AUC than TyG and TyG-BMI and a comparable AUC to TyG-WC, suggesting moderate discriminative ability for incident DM. Consistent with this, time-dependent ROC assessment across a prediction horizon spanning 2.0 to 5.0 years suggested that the AUC of TyGHR remained broadly stable within the range of 0.7351–0.7402, which may reflect a relatively sustained discriminative performance for DM incidence over both short- and extended follow-up windows. On the basis of these findings, TyGHR — a straightforward, readily obtainable, and methodologically reproducible composite biomarker — may hold potential as a useful supplementary indicator for anticipating DM occurrence, with possible utility in the earlier identification of at-risk individuals and the guidance of preventive decision-making. That said, further validation through prospective studies conducted in diverse, independent cohorts would be necessary before the clinical applicability of TyGHR in risk stratification can be more definitively established.

TyGHR and the precise pathophysiological mechanisms underlying DM onset have not been fully elucidated; however, available evidence suggests that they may be closely related to IR. The TyG components reflect the dual metabolic burden of IR and lipid excess. Elevated FPG reflects impaired insulin-mediated suppression of hepatic gluconeogenesis, while hypertriglyceridemia indicates increased production and decreased clearance of very-low-density lipoprotein (VLDL), and both are important features of IR (53, 54). Further, lipid intermediate metabolites such as diacylglycerol and ceramides accumulate in insulin-sensitive tissues, activating protein kinase C (PKC) isoforms and the c-Jun N-terminal kinase (JNK) pathway, leading to serine phosphorylation of insulin receptor substrate (IRS), thereby weakening insulin signal transduction and aggravating IR (55). In contrast, HDL-c, in addition to participating in reverse cholesterol transport, also has pleiotropic effects. It can promote cholesterol efflux via ATP-binding cassette transporter A1 (ABCA1) and G1 (ABCG1), thereby reducing intracellular cholesterol accumulation and alleviating endoplasmic reticulum stress—processes that are essential for maintaining insulin sensitivity (22, 23). In addition, HDL-c also possesses anti-inflammatory and antioxidative properties, partly mediated through related enzymes such as paraoxonase-1 (PON1), which can inhibit endothelial cell activation and reduce oxidative stress in adipose tissue and skeletal muscle (48). Therefore, decreased HDL-c may not only hinder cholesterol efflux but also enhance low-grade systemic inflammation, further worsening IR. Thus, TyGHR may comprehensively reflect the interaction and pathophysiological links among dysregulated glucose–lipid metabolism, chronic inflammation, and impaired insulin signal transduction.

In addition, restricted cubic spline modeling within the Cox proportional hazards framework was applied to assess whether the association between TyGHR and incident DM followed a nonlinear pattern. The analysis indicated a curvilinear relationship, with an estimated statistical threshold of 7.27. Below this threshold, each 1-unit increase in TyGHR was associated with a 29.6% higher risk of incident DM. However, the association above this threshold did not reach statistical significance. This nonsignificant finding should be interpreted cautiously and should not be regarded as evidence of no association. It may be influenced by the smaller number of participants and incident DM events in the higher TyGHR range, the sparse distribution of high TyGHR values, and potential instability of model estimates at the upper end of the exposure distribution. Further comparison showed that participants with TyGHR ≥7.27 had less favorable metabolic profiles, including higher blood pressure, BMI, HbA1c, liver enzymes, inflammatory markers, and lipid-related indicators, than those with TyGHR <7.27 (**Supplementary Table 7**). These baseline differences suggest that individuals in the higher TyGHR range may have a more complex metabolic risk background, which may contribute to greater uncertainty in estimating the independent association between TyGHR and incident DM in this subgroup. From a clinical perspective, the nonlinear pattern suggests that TyGHR may help identify individuals at increased metabolic risk before overt DM develops, particularly among those with rising TyGHR levels below the estimated threshold. As TyGHR is derived from routinely measured fasting glucose, triglycerides, and HDL-c, it may serve as a simple and accessible marker for early DM risk stratification and for guiding closer metabolic monitoring and lifestyle-related risk management. Nevertheless, the estimated threshold should be interpreted as a statistical inflection point rather than a causal intervention target. Future prospective studies are needed to verify this nonlinear pattern and to determine whether TyGHR-guided risk stratification can improve early prevention strategies for DM.

This investigation has several notable methodological strengths. To the best of our knowledge, this investigation may represent one of the earliest efforts to characterize the association between TyGHR and DM susceptibility within a Chinese cohort. TyGHR was evaluated both continuously and after categorization, which helped preserve quantitative information while allowing assessment of potential risk gradients, thereby providing a more complete characterization of its association with DM. Second, the present analysis identified a nonlinear association between TyGHR and subsequent DM occurrence and further determined a threshold value, adding new evidence to the understanding of this relationship. Third, incomplete covariate information was handled through multiple imputation, a strategy that enhances statistical efficiency while minimizing the systematic bias that may arise from missing data. Fourth, the robustness of the observed associations was evaluated via a comprehensive battery of sensitivity analyses: TyGHR was categorized into discrete strata for supplementary examination; nonlinear relationships between continuous covariates and the outcome were accommodated using smooth spline terms within a generalized additive modeling framework; E-values were computed to quantify the degree to which unmeasured confounding would need to be present to nullify the primary results; and the core analyses were repeated following the sequential exclusion of participants with obesity, hypertension, or a history of alcohol use.

Several limitations should be acknowledged. First, because the analytic sample consisted exclusively of Chinese participants, extrapolation of the results to other ethnicities or regions should be made with caution. External validation in populations with greater demographic and geographic diversity is therefore required. Second, due to the annual frequency of health examinations, interval censoring is an inherent limitation of this study: the true onset of DM may occur at any point between the last negative and the first positive examination. However, the sensitivity analysis — in which the DM event date was reassigned to the midpoint between these two examinations — yielded results consistent with the primary analysis. Furthermore, time-dependent ROC analysis demonstrated that the AUC of TyGHR for predicting incident DM ranged from 0.7351 to 0.7402 across follow-up intervals of 2 to 5 years, with AUC values remaining stable across time points, indirectly suggesting that minor variations in event date definition do not materially affect the overall conclusions of this study. Third, TyGHR and related metabolic indicators were measured only at baseline, and repeated assessments during follow-up were unavailable. Consequently, the present analysis could not evaluate temporal variations in TyGHR or determine how such changes might relate to subsequent DM occurrence. Future investigations incorporating serial measurements are needed to clarify the prognostic significance of dynamic TyGHR trajectories. Fourth, Since the data in this study were derived from routine health examination records, several variables essential to established DM risk scoring systems—such as family history of DM and dietary habits required by tools such as FINDRISC—were unavailable, precluding direct comparison with such instruments in the present cohort. Future studies employing a prospective cohort design will incorporate a more comprehensive set of multidimensional variables to enable systematic comparative validation of TyGHR against established DM risk scoring systems. Fifth, due to the inherent limitations of the retrospective cohort design, adjustment for potential confounders, such as dietary habits, family history of DM, changes in medication regimens during follow-up, socioeconomic status, and fasting insulin levels, was somewhat limited. Although we calculated the E-value and inferred that these unmeasured confounders were unlikely to substantially affect the association between TyGHR and DM, this interpretation is based only on statistical evidence and cannot completely exclude their potential influence on the results. Future prospective cohort studies with more comprehensive information on confounding factors are needed to further validate these findings. Finally, as with all observational studies, the present study can demonstrate an independent association between TyGHR and incident DM but cannot establish causality. Future multicenter prospective cohort studies are needed to further validate these findings and clarify the temporal relationship between TyGHR and incident DM.

## Conclusion

TyGHR was independently and positively associated with incident DM and showed a nonlinear pattern with an estimated threshold of approximately 7.27. Its relatively stable predictive performance over 2 to 5 years suggests potential value as a simple marker for early DM risk stratification and preventive strategies. Further prospective studies and external validation are warranted before routine clinical application.

## Data Availability

The raw data supporting the conclusions of this article will be made available by the authors, without undue reservation.
