# Supplementary material for: Association of the triglyceride-glucose index/high-density lipoprotein cholesterol ratio, a novel insulin resistance marker, with incident diabetes mellitus: a large-scale retrospective cohort study
Source: Front Endocrinol (Lausanne). 2026 Aug 10;17:1896239. doi: 10.3389/fendo.2026.1896239 (PMC13500281; doi:10.3389/fendo.2026.1896239)
Supplement: Supplementary file 1 [file DataSheet1.docx]

Table S1 VIF multicollinearity screening

|  | Step 1 | Step 2 | Step 3 | Step 4 | Step 5 |
| --- | --- | --- | --- | --- | --- |
| TyGHR | 11.2 | 11.2 | 10.9 | 2 | 2 |
| Age | 1.3 | 1.3 | 1.3 | 1.3 | 1.3 |
| HTN | 4.5 | 4.5 | 4.5 | 4.5 | 4.5 |
| DLP | 1.3 | 1.3 | 1.3 | 1.3 | 1.3 |
| DLP_MED | 1.3 | 1.3 | 1.3 | 1.3 | 1.3 |
| HTN_MED | 4.5 | 4.5 | 4.4 | 4.4 | 4.4 |
| SBP | 3.2 | 3.2 | 3.2 | 3.2 | 3.2 |
| DBP | 3.1 | 3.1 | 3.1 | 3.1 | 3.1 |
| BMI | 4.3 | 4.3 | 4.3 | 4.3 | 1.6 |
| WC | 5.3 | 5.3 | 5.3 | 5.3 | NA |
| Smoking | 1 | 1 | 1 | 1 | 1 |
| TC | 77.1 | NA | NA | NA | NA |
| LDL | 65.1 | 1.3 | 1.2 | 1.2 | 1.2 |
| AST | 1.9 | 1.9 | 1.9 | 1.9 | 1.9 |
| ALT | 2.5 | 2.5 | 2.5 | 2.5 | 2.5 |
| CRP | 1 | 1 | 1 | 1 | 1 |
| GGT | 1.4 | 1.4 | 1.4 | 1.4 | 1.4 |
| Physical activity | 1.1 | 1.1 | 1.1 | 1.1 | 1.1 |
| Drinking | 1.5 | 1.5 | 1.5 | 1.5 | 1.5 |
| Sex | 2 | 2 | 2 | 1.9 | 1.6 |

Variables Eliminated by Multicollinearity Screening: WC and TC.

Table S2. Comparison of Restricted Cubic Spline (RCS) Models for the Relationship between TyGHR and DM.

| Model | Knot Percentiles | Knot Values (TyGHR) | AIC | BIC | P value (vs Linear) | Model Fit Interpretation |
| --- | --- | --- | --- | --- | --- | --- |
| Linear | – | – | 5243.20 | 5287.31 | – | Baseline model |
| RCS (3 knots) | 5, 50, 95 | 4.228, 6.935, 10.916 | 5244.83 | 5278.34 | 0.082 | No statistically significant improvement |
| RCS (4 knots) | 5, 35, 65, 95 | 4.228, 6.181, 7.796, 10.916 | 5242.93 | 5250.28 | 0.007 | Best fit (lowest AIC/BIC) |
| RCS (5 knots) | 5, 25, 50, 75, 95 | 4.228, 5.646, 6.935, 8.467, 10.916 | 5243.48 | 5293.04 | 0.038 | Comparable to 4-knot model |

Table S3. A sensitivity analysis in which the timing of DM occurrence is redefined as the midpoint between the date of the last normal physical examination and the date of the current abnormal physical examination

| Exposure | HR,95%CI, P |
| --- | --- |
| TyGHR (Per 1-unit) | 1.067 (1.007, 1.130) 0.028 |
| TyGHR (quartiles) |  |
| Q1 | Ref |
| Q2 | 1.140 (0.753, 1.728) 0.535 |
| Q3 | 1.429 (0.957, 2.136) 0.081 |
| Q4 | 1.532 (1.027, 2.286) 0.037 |
| P for trend | 0.018 |

Adjust age, drinking status, sex, BMI, ALT, LDL-c, physical activity, HbA1c, DBP, smoking status, Scr, CRP, GGT, DLP-MED, HTN, and SBP.

HR, hazard ratio;Ref: reference; CI: confidence.

Table S4 Comparison of baseline characteristics between included and excluded participants

| Variables | Included participants | excluded participants | P-value |
| --- | --- | --- | --- |
| Age (years) | 41.82 ± 8.60 | 41.54 ± 9.66 | 0.078 |
| SBP (mmHg) | 116.99 ± 12.58 | 117.13 ± 12.86 | 0.593 |
| DBP (mmHg) | 76.10 ± 8.11 | 76.31 ± 8.30 | 0.060 |
| BMI (kg/m²) | 26.07 ± 3.87 | 26.12 ± 4.27 | 0.321 |
| WC (cm) | 91.19 ± 12.17 | 91.23 ± 12.91 | 0.691 |
| LDL-c (mg/dL) | 122.31 ± 33.50 | 122.74 ± 34.27 | 0.428 |
| TC (mg/dL) | 197.44 ± 36.75 | 198.45 ± 38.14 | 0.148 |
| HbA1c (mmol/L) | 5.00 ± 0.26 | 5.00 ± 0.26 | 0.942 |
| AST (U/L) | 29.27 ± 11.59 | 29.26 ± 13.12 | 0.980 |
| ALT (U/L) | 35.00 (27.00–47.00) | 35.12 (26.8–45.90) | 0.121 |
| CRP (mg/L) | 1.10 (0.60–2.40) | 1.11 (0.61–2.30) | 0.071 |
| GGT (U/L) | 27.00 (19.00–39.00) | 26.99 (19.01–38.97) | 0.137 |
| Hypertension (HTN), n (%) | | | |
| No | 7,903 (89.92%) | 13,251 (89.08%) | 0.079 |
| Yes | 886 (10.08%) | 1,625 (10.92%) |  |
| HTN_MED, n (%) | | | |
| No | 7,909 (89.99%) | 13,262 (89.15%) | 0.066 |
| Yes | 880 (10.01%) | 1,614 (10.85%) |  |
| Smoking n (%) | | | |
| No | 6,151 (69.99%) | 10,555 (70.95%) | 0.092 |
| Yes | 2,638 (30.01%) | 4,321 (29.05%) |  |
| Physical Activity, n (%) | | | |
| Sedentary | 1,899 (21.61%) | 3,398 (22.84%) | 0.051 |
| Light activity | 3,334 (37.93%) | 5,643 (37.93%) |  |
| Moderate activity | 2,790 (31.74%) | 4,605 (30.96%) |  |
| Vigorous activity | 766 (8.72%) | 1,230 (8.27%) |  |

Abbreviations: WC,waist circumference; TyGHR, Triglyceride-glucose index-to-HDL-c ratio;GGT, γ-glutamyl transferase; GHR, the ratio of γ-glutamyl transferase to high-density lipoprotein cholesterol; HbA1c,Hemoglobin A1c; LDL-c, low-density lipid cholesterol; SBP, systolic blood pressure; TG triglyceride; BMI, body mass index; TC, total cholesterol, ALT, alanine aminotransferase; DBP, diastolic blood pressure; AST aspartate aminotransferase.

Hypertension (HTN), n (%)

No	7,903 (89.92%)	13,251 (89.08%)

Yes	886 (10.08%)	1,625 (10.92%)

P = 0.053

HTN_MED, n (%)

No	7,909 (89.99%)	13,262 (89.15%)

Yes	880 (10.01%)	1,614 (10.85%)

P = 0.050

Smoking, n (%)

No	6,151 (69.99%)	10,555 (70.95%)

Yes	2,638 (30.01%)	4,321 (29.05%)

P = 0.078

Physical Activity, n (%)

Sedentary	1,899 (21.61%)	3,398 (22.84%)

Light activity	3,334 (37.93%)	5,643 (37.93%)

Moderate activity 2,790 (31.74%)	4,605 (30.96%)

Vigorous activity	766 (8.72%)	1,230 (8.27%)

P = 0.051

Table S5 Stratified associations between TyGHR and the risk of DM by age, sex, BMI, ASBP, DBP, smoking status, HTN-MED, DLP, and Physical Activity

| Characteristics | participants | HR (95%CI) *P* value | *P* for interaction |
| --- | --- | --- | --- |
| Age(years) |  |  | 0.7281 |
| <30 | 454 | 1.088 (0.974, 1.215) 0.1367 |  |
| 30-40 | 3278 | 1.088 (0.974, 1.215) 0.1367 |  |
| 40-50 | 3337 | 1.092 (1.006, 1.185) 0.0359 |  |
| 50-60 | 1485 | 1.150 (1.042, 1.269) 0.0056 |  |
| ≥60 | 235 | 1.114 (0.888, 1.398) 0.3504 |  |
| Sex |  |  | 0.6194 |
| Female | 3218 | 1.145 (0.994, 1.319) 0.0613 |  |
| Male | 5571 | 1.102 (1.036, 1.171) 0.0020 |  |
| Smoking |  |  | 0.1580 |
| No | 8109 | 1.097 (1.034, 1.164) 0.0023 |  |
| Yes | 680 | 1.272 (1.043, 1.551) 0.0176 |  |
| BMI categories |  |  | 0.5830 |
| <18.5 kg/m^2^ | 588 | 1.105 (1.008, 1.212) 0.0335 |  |
| 18.5-24 kg/m^2^ | 5932 | 1.105 (1.008, 1.212) 0.0335 |  |
| 24-28 kg/m^2^ | 1049 | 1.125 (1.009, 1.254) 0.0333 |  |
| ≥28.0kg/m^2^ | 1220 | 1.107 (1.012, 1.210) 0.0259 |  |
| SBP |  |  | 0.4003 |
| <140 mmHg | 5992 | 1.118 (1.052, 1.189) 0.0004 |  |
| ≥140mmHg | 282 | 1.050 (0.915, 1.205) 0.4863 |  |
| DBP |  |  | 0.356 |
| <90 mmHg | 8049 | 1.109 (1.042, 1.180) 0.0011 |  |
| ≥90mmHg | 740 | 1.092 (0.955, 1.249) 0.1973 |  |
| HTN-MED |  |  | 0.2847 |
| No | 7909 | 1.090 (1.021, 1.163) 0.0096 |  |
| Yes | 880 | 1.164 (1.046, 1.296) 0.0055 |  |
| DLP |  |  | 0.3233 |
| No | 6387 | 1.120 (1.042, 1.205) 0.0021 |  |
| Yes | 2402 | 1.063 (0.978, 1.156) 0.1527 |  |
| Physical Activity |  |  | 0.7471 |
| Sedentary | 1899 | 1.134 (1.031, 1.248) 0.0100 |  |
| Low | 3334 | 1.113 (1.023, 1.212) 0.0130 |  |
| Moderate | 2790 | 1.053 (0.943, 1.177) 0.3563 |  |
| High | 766 | 1.144 (0.935, 1.399) 0.1914 |  |

Note 1: Above model adjusted for age, sex, BMI, GGT, SBP, TG, ALT, HS-CRP, HbA1c, current smoking, physical activity, HTN, DM, DM-MED, DLP-MED, HTN-MED.

Note 2: In each case, the model is not adjusted for the stratification variable.

Abbreviations: HR, Hazard ratios; CI: confidence, Ref: reference; SBP, systolic blood pressure; DBP, diastolic blood pressure; DM-MED, antidiabetic medication; DLP, Dyslipidemia. AST, Aspartate Aminotransferase; BMI, Body Mass Index; GGT, Gamma-Glutamyl Transferase; ALT,Alanine Aminotransferase.

Table S6. Time-dependent ROC curves of baseline TyGHR for predicting incident DM within 2.0, 3.0, 4.0, and 5.0 years.

| Follow-up (years) | AUC (95% CI) | Best threshold | Specificity | Sensitivity | Youden Index |
| --- | --- | --- | --- | --- | --- |
| 2.0 | 0.7351 (0.6994–0.7708) | 6.825 | 0.7639 | 0.6196 | 0.3835 |
| 3.0 | 0.7401 (0.7101–0.7701) | 6.735 | 0.7670 | 0.6181 | 0.3851 |
| 4.0 | 0.7402 (0.7117–0.7687) | 6.741 | 0.7616 | 0.6224 | 0.3840 |
| 5.0 | 0.7389 (0.7116–0.7661) | 6.560 | 0.7697 | 0.6151 | 0.3848 |

Abbreviations: AUC: area under the curve; CI: Confidence interval.

Table S7 Comparison of baseline characteristics between participants with TyGHR <7.27 and TyGHR ≥7.27.

| Variables | TyGHR <7.27 | TyGHR >=7.27 | P-value |
| --- | --- | --- | --- |
| N | 4941 | 3848 |  |
| Age(years) | 41.404 ± 8.735 | 42.350 ± 8.387 | <0.001 |
| SBP (mmHg) | 114.355 ± 12.021 | 120.375 ± 12.464 | <0.001 |
| DBP (mmHg) | 74.379 ± 7.880 | 78.310 ± 7.861 | <0.001 |
| WC(cm) | 87.937 ± 11.731 | 96.916 ± 11.187 | <0.001 |
| BMI (kg/m2) | 24.963 ± 3.485 | 27.495 ± 3.868 | <0.001 |
| TC (mg/dL) | 195.016 ± 35.254 | 200.549 ± 38.378 | <0.001 |
| LDL-c(mg/dL) | 118.375 ± 32.939 | 129.912 ± 33.632 | <0.001 |
| TG(mg/dL) | 95.290 ± 39.470 | 164.166 ± 80.551 | <0.001 |
| AST(u/L) | 27.816 ± 10.690 | 31.140 ± 12.414 | <0.001 |
| ALT(u/L) | 34.841 ± 17.156 | 46.134 ± 22.621 | <0.001 |
| CRP (mg/dL) | 2.258 ± 5.020 | 2.551 ± 4.998 | 0.007 |
| GGT(u/L) | 29.443 ± 23.965 | 38.945 ± 27.418 | <0.001 |
| Sex |  |  | <0.001 |
| Female | 1934 (39.142%) | 284 (7.380%) |  |
| Male | 3007 (60.858%) | 3564 (92.620%) |  |
| HTN(n,%) | 379 (7.671%) | 507 (13.176%) | <0.001 |
| Smoking(n,%) | 315 (6.375%) | 365 (9.485%) | <0.001 |
| Physical Activity(n,%) |  |  | <0.001 |
| Sedentary | 928 (18.782%) | 971 (25.234%) |  |
| Light activity | 1830 (37.037%) | 1504 (39.085%) |  |
| Moderate activity | 1659 (33.576%) | 1131 (29.392%) |  |
| Vigorous activity | 524 (10.605%) | 242 (6.289%) |  |
| Drinking(n,%) | 845 (17.102%) | 1793 (46.596%) | <0.001 |

Abbreviations: WC,**waist circumference;** TyGHR, Triglyceride-glucose index-to-HDL-c ratio;GGT, γ-glutamyl transferase; GHR, the ratio of γ-glutamyl transferase to high-density lipoprotein cholesterol; HbA1c,**Hemoglobin A1c;** LDL-c, low-density lipid cholesterol; SBP, systolic blood pressure; TG triglyceride; BMI, body mass index; TC, total cholesterol, ALT, alanine aminotransferase; DBP, diastolic blood pressure; AST aspartate aminotransferase

**Figure S1 ROC Curve of TyGHR for Predicting DM with Bootstrap Validation (500 Iterations)**

**Figure S2 Decision Curve Analysis (DCA):** It presents the standardized net benefit of TyGHR across various thresholds in predicting DM, including cost-benefit ratios.

**Figure S3 Calibration Curve:** This plot illustrates the calibration of TyGHR for DM prediction, showing the relationship between predicted and observed probabilities.
